# Draft Genome Sequences of Two Isolates of *Colletotrichum lindemuthianum*, the Causal Agent of Anthracnose in Common Beans

**DOI:** 10.1128/genomeA.00214-17

**Published:** 2017-05-04

**Authors:** Casley Borges de Queiroz, Hilberty L. Nunes Correia, Renato Pedrozo Menicucci, Pedro M. Pereira Vidigal, Marisa Vieira de Queiroz

**Affiliations:** a Departamento de Microbiologia, Instituto de Biotecnologia Aplicada a Agropecuária (BIOAGRO), Universidade Federal de Viçosa, Minas Gerais, Brazil; b Núcleo de Análise de Biomoléculas (NuBioMol), Centro de Ciências Biológicas, Universidade Federal de Viçosa, Minas Gerais, Brazil

## Abstract

*Colletotrichum lindemuthianum* is the causal agent of anthracnose in common beans, one of the main limiting factors of their culture. Here, we report for the first time, to our knowledge, a draft of the complete genome sequences of two isolates belonging to 83.501 and 89 A_2_ 2-3 of *C. lindemutuianum*.

## GENOME ANNOUNCEMENT

*Colletotrichum* is one of the most widespread and economically important genera among the various damaging plant pathogens (1). This is especially true in tropical and subtropical regions, where *Colletotrichum* spp. limit the production of important crops, causing severe social and economic impacts (2). *Colletotrichum lindemuthianum* is the causal agent of anthracnose in the common bean (*Phaseolus vulgaris* L.) and attacks its leaves, stems, branches, string beans, and seeds (3). Under favorable conditions of humidity and temperature, in extreme cases, anthracnose can cause death of the plant (4). *C. lindemuthianum* has a high genetic variability, manifested by the presence of many physiologically diverse races (5). This high genetic variability is one of the main limiting factors to combating this pathogen, because it prevents the long-term use of resistant cultivars (6). The genomes of two isolates, 83.501 and 89 A_2_ 2-3, of *C. lindemuthianum* were sequenced using the HiSeq 2500 Illumina platform with paired-end reads of 100 bp and an average coverage of 89.3× for isolate 83.501 and 157.7× for isolate 89 A_2_ 2-3 (Laboratório Central de Tecnologias de Alto Desempenho em Ciências de Vida [LaCTAD]). The genes were predicted using Augustus version 3.2.2 (7). For *de novo* assembly, we used CLC Genomics Workbench 6.5.1. The completeness of the genome was estimated using Benchmarking Universal Single-Copy Orthologs (BUSCO) (8).

The contig assembly of isolate 83.501 using the CLC Genomics Workbench software resulted in 1,857 contigs (*N*_50_, 111.275 kbp), the smallest containing a 1.02-kbp band and the largest containing a 623.12-kbp band, for a total of 97.4 Mbp and a G+C content of 37.6%. The contig assembly of isolate 89 A_2_ 2-3 resulted in 1,276 contigs (*N*_50_, 158.217 kb), the smallest containing 1.03 kbp and the largest containing 1.11 Mbp, for a total of 99.16 Mbp, with a G+C content of 37.3%. Assessment of the completeness of the genome using 1,438 BUSCO groups for fungi resulted in 98% complete (C) (6.1% duplicated [D]), 1.0% fragmented (F), 0.2% missed (M), and 1,438 genes (*n*) for isolate 83.501 and 98% C (6.0% D), 1.1% F, 0.4% M, and 1,438 *n* for isolate 89 A_2_ 2-3, indicating that the assembled genome covered most of the coding regions. There were 11,673 (isolate 83.501) and 11,627 (isolate 89 A_2_ 2-3) predicted genes.

In this study, we present a draft of the genome sequences belonging to two isolates from different races of *C. lindemuthianum*. This information will provide a valuable resource for identifying the mechanisms of pathogenesis and the plant-pathogen interactions, as well as new pathogenicity factors. Our data will also allow for comparative genomic studies with other fungal plant pathogens.

### Accession number(s).

The sequences from this whole-genome shotgun project for the two strains have been deposited in the DDBJ/ENA/GenBank database under accession numbers MASO00000000 and MASP00000000. The versions described in this paper are the first versions.
